# α-Spectrin and integrins act together to regulate actomyosin and columnarization, and to maintain a monolayered follicular epithelium

**DOI:** 10.1242/dev.130070

**Published:** 2016-04-15

**Authors:** Bing Fu Ng, Gokul Kannan Selvaraj, Carmen Santa-Cruz Mateos, Inna Grosheva, Ines Alvarez-Garcia, María Dolores Martín-Bermudo, Isabel M. Palacios

**Affiliations:** 1 Department of Zoology, University of Cambridge, Downing Street, Cambridge CB2 3EJ, UK; 2 Centro Andaluz de Biología del Desarrollo CSIC-Univ. Pablo de Olavide, Sevilla 41013, Spain

**Keywords:** Tissue architecture, Epithelium, Monolayer, Tumor-like mass, Proliferation, Cell shape

## Abstract

The spectrin cytoskeleton crosslinks actin to the membrane, and although it has been greatly studied in erythrocytes, much is unknown about its function in epithelia. We have studied the role of spectrins during epithelia morphogenesis using the *Drosophila* follicular epithelium (FE). As previously described, we show that α-Spectrin and β-Spectrin are essential to maintain a monolayered FE, but, contrary to previous work, spectrins are not required to control proliferation. Furthermore, spectrin mutant cells show differentiation and polarity defects only in the ectopic layers of stratified epithelia, similar to integrin mutants. Our results identify α-Spectrin and integrins as novel regulators of apical constriction-independent cell elongation, as *α-Spectrin* and integrin mutant cells fail to columnarize. Finally, we show that increasing and reducing the activity of the Rho1-Myosin II pathway enhances and decreases multilayering of **α-*S*pectrin** cells, respectively. Similarly, higher Myosin II activity enhances the integrin multilayering phenotype. This work identifies a primary role for α-Spectrin in controlling cell shape, perhaps by modulating actomyosin. In summary, we suggest that a functional spectrin-integrin complex is essential to balance adequate forces, in order to maintain a monolayered epithelium.

## Abstract (precis)

**Summary:** α-Spectrin and integrins regulate apical constriction-independent cell elongation, identifying a primary role for α-Spectrin in controlling cell shape.

## INTRODUCTION

Monolayered epithelia are sheets of adherent, polarized cells that act as physical barriers and constitute structural components of organs and tissues. The formation and maintenance of the monolayered structure are crucial for both proper function of the epithelia and whole-body homeostasis. During carcinogenesis, loss of epithelial architecture leads to the formation of multilayered epithelia, disorganized cell masses and increased tumorigenic potential.

**Fig. 1.**
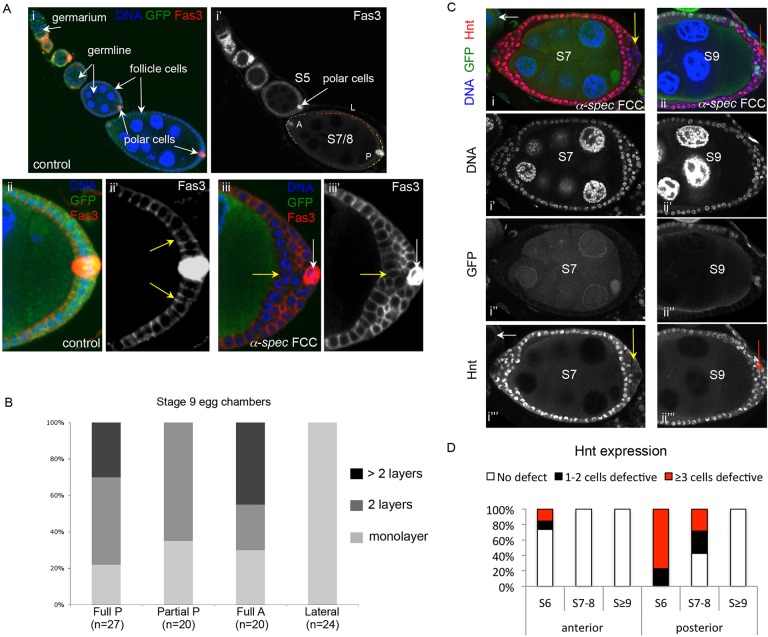
***α-Spectrin* mutant epithelia form multilayers with aberrant Hindsight and Fasciclin 3 expression only in ectopic layers.** (A) Fas3 is expressed in the FE in early oogenesis (i,i′, egg chambers budding off of the germarium), but becomes restricted to the polar cells by S5 (i′,ii). When *α-Spec* cells (follicle cell clones, FCCs) form a multilayer (iii), cells of the ectopic layers express a Fas3 level intermediate to those of the germline-adjoining FCs (yellow arrow) and the polar cells (white arrows) (*n*=15). Note in ii the accumulation of Fas3 in an apicolateral region (yellow arrows in ii′), which is relevant for later findings. (i′) From S7, the epithelium is divided into lateral (L, red line), anterior (A, white line) and posterior (P, yellow line) domains. The posterior FC (PFC) domain is formed by a cap of ∼200 cells surrounding the polar cells, and correlates in the cross-sections presented in most figures with a region that expands by ten cells to each side of the polar cells. Panels ii-iii′ show the posterior domain. Fas3 is in red in merge panels. (B) Quantification reveals that epithelial stratification is never observed in *α-Spec* mutant lateral epithelia (Lateral), being limited to the terminal (anterior, A, or posterior, P) domains only. Epithelial integrity is compromised even when *α-Spec* clones make up part of the posterior (Partial P clone). (C) Hnt is not expressed before S6 of oogenesis (white arrows in i and i‴). After S6 the Notch pathway activates Hnt expression. This Notch-dependent upregulation of Hnt is often defective in the ectopic layers of S6-8 *α-Spec* multilayered epithelia (yellow arrows in i and i‴). However, Hnt upregulation is normal by S9, even in ectopic layers (red arrows in ii and ii‴; see also D). The egg chambers are positioned with the anterior to the left. Hnt is in red in merge panels. (D) Quantification of mosaic egg chambers containing control and *α-Spec* mutant cells reveals that young egg chambers (S6-8) have severe Hnt expression defects (at least three cells defective), whereas older egg chambers (S9 and beyond) are non-defective. This trend is more pronounced at the posterior. *n*=45, 32 and 24 for S6, S7-8 and S≥9, respectively. In A and C, merged images show DAPI in blue, and mutant cells lack GFP.

The *Drosophila melanogaster* ovary constitutes an excellent model system in which to study the molecular and cellular basis of epithelial morphogenesis. The adult ovary is composed of various ovarioles that contain a line of egg chambers at different developmental stages [stage1-14 (S1-14)]. Each egg chamber is composed of 16 germline cells (including the oocyte), and a layer of somatic cells (the follicle cells, FCs) forming a monolayered epithelium termed the follicular epithelium (FE) (Fig. 1A). FCs are derived from stem cells that are located in the germarium. Up to S6 of oogenesis, FCs undergo several rounds of mitotic cycles to form the FE, then exit mitosis and enter an endocycle. From S7, most FCs change their shape from cuboidal to columnar, and migrate towards the posterior (Fig. S1). The factors important for formation of a monolayered FE are not yet fully understood, but mutations in genes controlling polarity and mitosis lead to FE multilayering, such as aPKC (Abdelilah-Seyfried et al., 2003), Notch and the Hippo pathway (Meignin et al., 2007; Polesello and Tapon, 2007; Yu et al., 2008). In addition, integrins and spectrins (Spec) are also important for maintaining a monolayer.

The spectrin-based membrane skeleton (SBMS) is a scaffold made from building blocks of tetramers of two α and two β Spec subunits that line the cell membrane. The function of the SBMS has been greatly studied in erythroid cells, and a variety of erythrocyte disorders are associated with mutations in Spec genes. Members of the Spec family are conserved in all eukaryotes, with a greater conservation between *Drosophila* and mammalian non-erythroid Specs than between erythroid and non-erythroid mammalian forms (Baines, 2003, 2009; Salomao et al., 2006). However, in contrast to mammals, the *Drosophila* genome features a single form of α-Spec (human αII-like), a conventional β subunit (human βII-like) and a heavy β subunit (β_H_), making it easier to characterize their function in non-erythroid cells (Byers et al., 1989; Dubreuil et al., 1990; Lee et al., 1997). The (αβ)_2_ and (αβ_H_)_2_ tetramers are distinctively localized in the basolateral and apical domains, respectively (Dubreuil et al., 1998; Lee et al., 1997; Thomas et al., 1998; Zarnescu and Thomas, 1999).

A diversity of functions has been attributed to spectrins based on studies in both cell culture and model organisms. In invertebrates, spectrins are essential for morphogenesis and animal growth. *Drosophila* spectrins have been recently identified as modulators of the cell growth Hippo pathway in various tissues (Deng et al., 2015; Fletcher et al., 2015; Wong et al., 2015). In ovaries, a mutant *β-Spec* allele with a premature stop codon at amino-acid 1046 shows defects in FE integrity, actin organization and oocyte polarity, partially phenocopying *hippo* mutants (Wong et al., 2015). *α-Spec* mutant FCs also form a stratified epithelium, with polarity defects (Lee et al., 1997), and FCs expressing an *α-Spec* RNAi show *hippo*-like differentiation defects (Fletcher et al., 2015). By contrast, *β_H_*-*Spec* does not regulate Hippo or actin in ovaries (Fletcher et al., 2015; Thomas et al., 1998; Zarnescu and Thomas, 1999). These findings confirm the idea that different tissues exhibit different dependence on the apical versus lateral spectrin cytoskeleton (Dubreuil et al., 2000; Hulsmeier et al., 2007; Thomas and Williams, 1999).

These previous studies concentrated on spectrins as Hippo modulators. We decided to broaden the analysis of SBMS function in a monolayered epithelium by studying the consequences of eliminating SBMS on the proliferation, polarization and differentiation of FCs, as well as on the architecture of the FE. As α-Spec is the major component of both the apical and lateral spectrin cytoskeletons, we decided to concentrate on *α-Spec*. Eliminating α-Spec in the FE results in stratification of the terminal regions, especially the posterior, supporting previous findings (Fletcher et al., 2015; Lee et al., 1997). However, in contrast to Hippo, we find that the function of the SBMS in the monolayered FE is not to control mitosis, differentiation or polarity, but to regulate the actomyosin cytoskeleton, septate junctions (SJs) and cell shape. The *α-Spec* mutant phenotype is similar to that of integrin [*myospheroid* (*mys*)] mutants, and α-Spec and integrins colocalize in the lateral membrane of the FCs. We propose that a functional spectrin-integrin complex is important for regulation of the actomyosin cytoskeleton and tissue architecture.

## RESULTS

### *α-Spectrin* mutant cells show differentiation defects only when forming a stratified epithelium

To understand the defects of eliminating *α-Spec* on FE morphogenesis, we studied FCs mutant for the null allele *α-Spec*^*rg41*^. As in previous reports, we found that *α-Spec* FCs form a multilayered epithelium. These tumor-like masses are observed only at the terminal (anterior/posterior) domains of the egg chamber, and never at the mid-body (Fig. 1). This terminal requirement for α-Spec in epithelial architecture is identical to the terminal requirement for Hippo and integrins (Fernández-Miñán et al., 2007; Meignin et al., 2007; Polesello and Tapon, 2007).

Mutations that result in a multilayered FE often prevent the FCs from differentiating. FCs differentiate from S6, as revealed by differential expression of markers, such as Fasciclin 3 (Fas3), Hindsight (Hnt; also known as Pebbled) and Eyes absent (Eya). Fas3 is expressed at high levels in immature FCs, but its expression becomes gradually restricted to the polar cells as oogenesis proceeds (Bai, 2002; Muzzopappa and Wappner, 2005) (Fig. 1Ai), whereas Hnt is upregulated from S6 (Sun and Deng, 2007). Analysis of mosaic FE containing both control and *α-Spec* mutant FCs (also named FCCs) revealed that some S6-8 *α-Spec* FCCs show high levels of Fas3 (Fig. 1Ai-Aii versus Aiii) and lack Hnt expression (Fig. 1Ci,D). However, in all cases, the defects are only observed in *α-Spec* cells located in the ectopic layers of the multilayered epithelium (Fig. 1A,C), and not in mutant cells that are either adjacent to the oocyte (Fig. 1Aiii, yellow arrow) or forming a monolayer (Fig. 5). This differentiation phenotype is stronger at the posterior than at the anterior pole, and weaker in older egg chambers: 100% (*n*=45) of the S6 *α-Spec* FCCs show Hnt defects, but these defects are absent at S9 (*n*=24) (Fig. 1C,D). Similarly, fewer *α-Spec* FCCs have defective Fas3 levels in older egg chambers (62.5% and 25% in early S6-8 and S9 egg chambers, respectively, *n*=16). It is interesting to note that even though Fas3 is properly downregulated in most *α-Spec* cells, the remaining Fas3 does not show the apicolateral accumulation that is observed in control posterior FCs (PFCs; Fig. 1Aii′ versus Aiii″, yellow arrows; Fig. 5; Dubreuil et al., 2001). Finally, Eya, which is downregulated in cells from S6, was also properly downregulated in *α-Spec* mutant monolayers, and *α-Spec* cells adjacent to the oocyte, but had a stronger expression in the *α-Spec* ectopic layers (data not shown).

Thus, α-Spec is required for the FE to maintain a monolayer, and for the FCs to mature only when part of ectopic layers. These varying defects in differentiation indicate that α-Spec plays a secondary role in this aspect of oogenesis, unlike the Hippo pathway, which fully blocks FC maturation when mutated in terminal FCs (Meignin et al., 2007; Polesello and Tapon, 2007).

### *α-Spectrin* mutant cells located in the ectopic layers show polarity defects

**Fig. 2.**
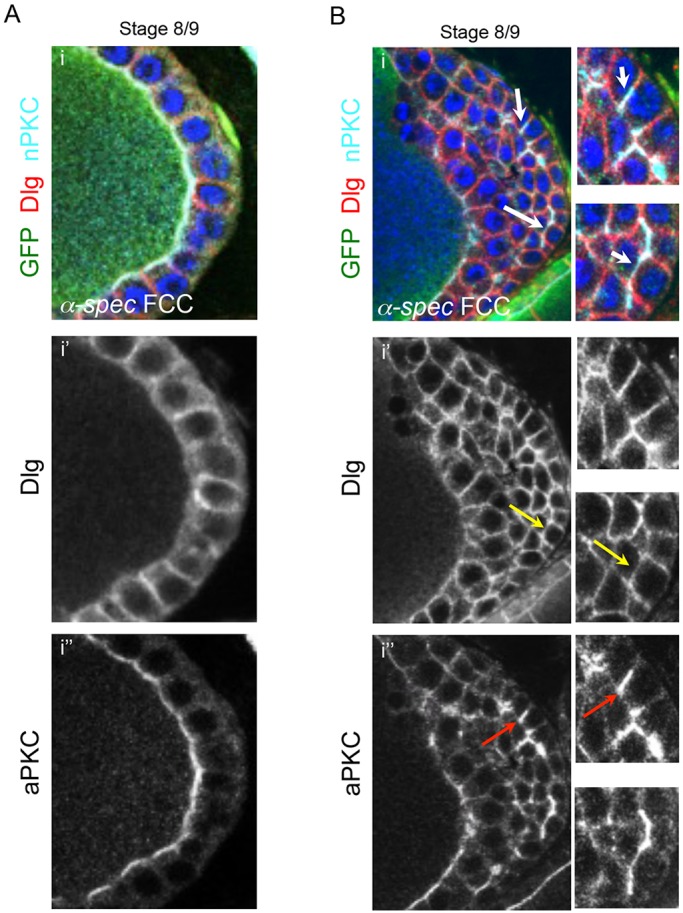
***α-Spectrin* mutant follicle cells display epithelial polarity defects in ectopic layers.** (A) The lateral marker Dlg and the apical marker aPKC localize correctly in *α-Spec* mutant cells that form a monolayer (*n*=12). (B) In a multilayered *α-Spec* mutant FE, correct localization of Dlg and aPKC is observed in germline-contacting FCs. In ectopic layers, Dlg is often mislocalized between ectopic layers of cells (i′, yellow arrows), and aPKC often expands into the lateral membrane (i″, red arrows). The white arrows in the top panel indicate colocalization between the mislocalized markers. Some degree of polarity is still preserved because Dlg and aPKC are generally localized correctly in *α-Spec* ectopic layers (*n*=35). All images show the posterior domain of S8/9 egg chambers. Blue, DAPI; red, Dlg; light blue, aPKC. *α-Spec* mutant clones (FCCs) lack GFP.

Recent work has shown that apical and lateral components [such as α-Spec, aPKC and Discs large (Dlg; Dlg1 – FlyBase)] localize properly in *β-Spec* mutant monolayers (Wong et al., 2015). By contrast, *α-Spec* FCs that are part of a hyperplastic region show a loss of epithelial polarity (Lee et al., 1997). However, the *α-Spec* ovaries analyzed by Lee et al. were also heterozygous for *discs lost* (*dlt*), a polarity gene. The *dlt* heterozygosity might have increased the polarity phenotype in *α-Spec* mutant FCs*.* To characterize further the role of *α-Spec* in epithelial polarity, we analyzed the distribution of aPKC and Dlg in FCs that lack only *α-Spec*. These proteins localize correctly in *α-Spec* FCCs that maintain a monolayered epithelium (Fig. 2A; *n*=15). However, mutant cells in ectopic layers show mislocalized aPKC and Dlg in 80% and 97% of the mutant epithelia, respectively (Fig. 2B; *n*=35). aPKC often expands into the lateral membrane (red arrows) and by S8-9 Dlg is no longer found restricted to the lateral membrane (yellow arrows), resulting in the colocalization of these two proteins in some instances (Fig. 2Bi, white arrows). However, some degree of polarity is still preserved, as Dlg and aPKC can localize correctly in the most ectopic layer.

All these observations suggest that spectrins become relevant for epithelial polarity when FCs lose contact with the germline or the basal membrane, or that an apical and basal cue can compensate for the lack of a spectrin cytoskeleton.

### Oocyte polarity is largely unaffected in egg chambers with *α-Spectrin* mutant follicle cells

The repolarization of the oocyte in mid-oogenesis is induced by a signal from the PFCs (Deng and Ruohola-Baker, 2000; Frydman and Spradling, 2001; González-Reyes et al., 1995; MacDougall et al., 2001; Roth et al., 1995), a process suggested to depend on the SBMS, as oocyte polarity is often aberrant in egg chambers with *β-Spec* clones (Wong et al., 2015) or *α-Spec, dlt*/+ cells (Lee et al., 1997). As we have observed that oocyte-adjacent *α-Spec* cells mature and polarize properly, we wondered whether elimination of only *α-Spec* affects repolarization of the oocyte. In wild-type egg chambers, oocyte nucleus migration from posterior to the dorsal-anterior corner is complete by S7, and provides a read-out of oocyte polarity. This migration is also observed in 95.5% of egg chambers with large *α-Spec* FCCs (Fig. S2; *n*=45). A more sensitive assay to detect oocyte polarity defects is Staufen localization, which by S9 forms a tight crescent at the posterior of the oocyte (Fig. S2A). Of the 95.5% mutant egg chambers that showed wild-type nucleus positioning, we found that Staufen is localized properly in all of them, whereas 13% of follicles with partial posterior clones show Staufen expressed in a crescent-shaped area that is shifted towards the control cells (Fig. S2B,D; data not shown). Thus, oocyte polarity is largely unaffected in egg chambers with *α-Spec* mutant PFCs.

### *α-Spectrin* is not required for cells to exit mitosis

**Fig. 3.**
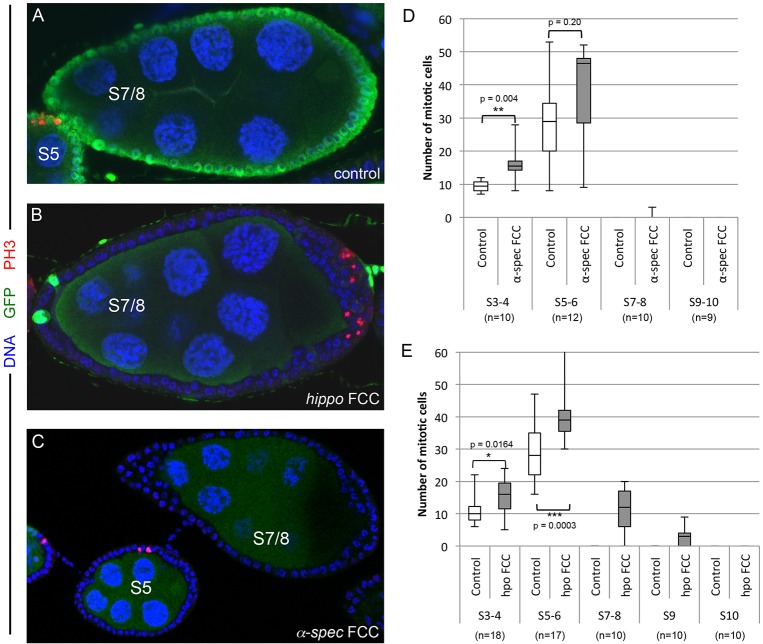
**Unlike the Hippo pathway, α-Spectrin is not required for follicle cells to exit mitosis.** (A-C) In contrast to *hippo* mutant cells (*hippo* FCCs, B), both control (A) and *α-Spec* FCCs (C) exit mitosis at S6 of oogenesis. (A) Control egg chambers showing PH3 (red) positive cells at S5, but not at S7/8. (B) S7/8 egg chamber with PH3 (red) positive *hippo* cells. (C) As in control, *α-Spec* FCCs are not positive for PH3 after S6 of oogenesis. Mutant cells lack GFP. Blue, DAPI. (D,E) Box plot quantification of mitotic cells (indicated by PH3 expression) in *α-Spec* (D) or *hippo* (E) S3-S10 egg chambers. S7-9 egg chambers containing *hippo* FCCs are positive for PH3. *α-Spec* FCCs are not positive for PH3 after S6, but they do display higher instances of PH3 in earlier stages (quantified in S3-6). *n* is as indicated for both the control and the mutant cells.

The results described above show that *α-Spec* FCCs show polarity and differentiation defects only in the ectopic layers of a multilayered epithelium, and induce mild oocyte polarity defects. By contrast, PFC differentiation and oocyte polarity is aberrant in all *hippo* mosaic egg chambers (Meignin et al., 2007; Polesello and Tapon, 2007; Yu et al., 2008). To test whether *α-Spec* FCCs show any *hippo*-like phenotype, we analyzed whether α-Spec is required for the FCs to exit mitosis, a process that is Hippo dependent (Meignin et al., 2007), by detecting phospho-histone 3/PH3. PH3 is only detected until S6, and never later, in control cells (Fig. 3A) (Deng et al., 2001; Schaeffer et al., 2004). By contrast, *hippo* FCs are often positive for PH3 at S7-10B (Fig. 3B,E) (Meignin et al., 2007; Polesello and Tapon, 2007). As with wild type, *α-Spec* FCCs never express PH3 after S6 (Fig. 3C,D). Hence, and unlike Hippo, α-Spec is not required for FCs to exit mitosis.

### Septate junction-components mislocalize in *α-Spectrin* mutant cells

Our initial observation that Fas3 accumulates apicolaterally in S9 PFCs (Fig. 1Aii′), and that such localization was lost in *α-Spec* cells in contact with the oocyte (Fig. 1Aiii′), suggest that α-Spec and Fas3 might be involved in processes other than FC maturation and polarity (as these processes are not aberrant in oocyte-adjacent *α-Spec* cells). Earlier works identified Fas3 as a component of SJs, the invertebrate counterparts of tight junctions (TJs) (Brower et al., 1980; Fehon et al., 1994; Patel et al., 1987; Woods and Bryant, 1991). SJs form basal to the zonula adherens (ZA), and in addition to blocking diffusion they also aid cell adhesion (Fehon et al., 1991; Tepass and Tanentzapf, 2001; Woods and Bryant, 1993; Zak and Shilo, 1992). The aberrant distribution of Fas3 in *α-Spec* FCs raises the possibility that α-Spec is required for SJs formation. Thus, we characterized the localization pattern during oogenesis of the SJ components Fas3, Dlg (Woods et al., 1996), Coracle (Cora) (Fehon et al., 1994) and Fas2 (Wei et al., 2004).

**Fig. 4.**
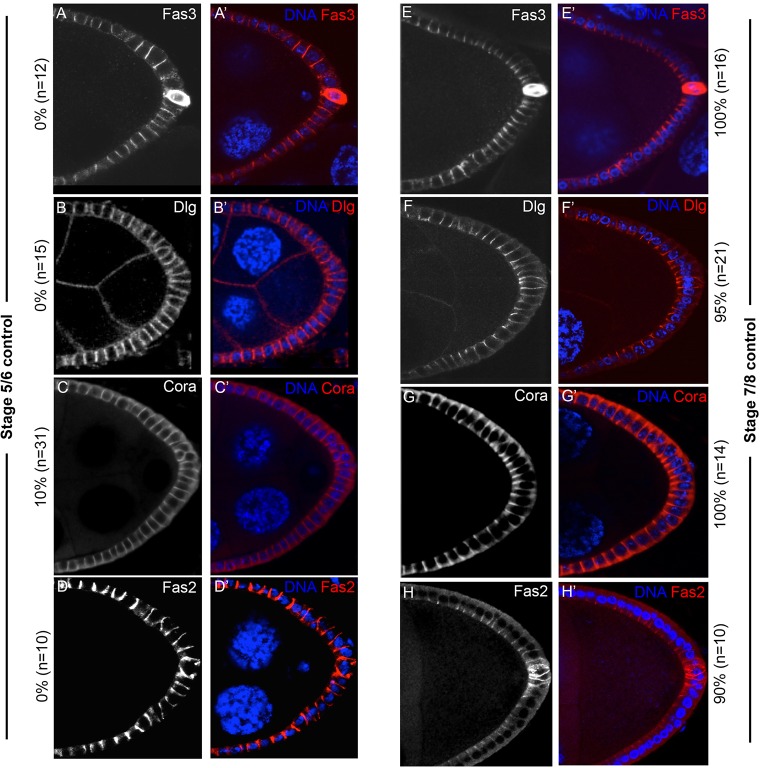
**Components of the septate junctions display similar localization patterns during oogenesis.** (A-D′) During early oogenesis, the septate junction (SJ) proteins Fas3 (A,A′), Dlg (B,B′), Coracle (Cora; C,C′) and Fas2 (D,D′) are expressed uniformly along the lateral membrane. (E-H′) From S7, an apicolateral concentration of Fas3 (E,E′), Dlg (F,F′), Cora (G,G′) and Fas2 (H,H′) becomes distinct, especially in the posterior FCs. The percentages indicate the frequency at which these SJ proteins localize apicolaterally. All merged images show DAPI in blue, and Fas3, Dlg, Fas2 and Cora in red.

**Fig. 5.**
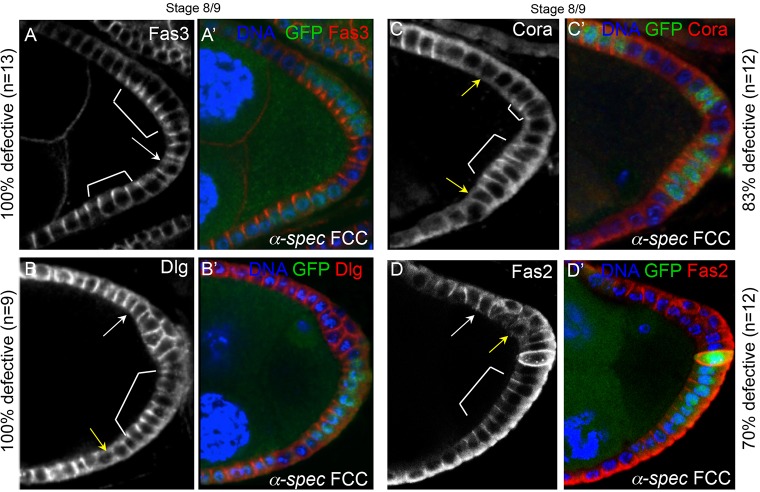
**The apicolateral localization of Fasciclin 3, Discs large, Coracle and Fasciclin 2 is lost in *α-Spectrin* mutant cells.** (A-D′) At S8/9, the apicolateral localization of the SJ proteins is lost in *α-Spec* mutant FCs (*α-Spec* FCCs) that form a monolayered FE (arrows), compared with their neighboring control FCs (brackets). The proteins sometimes extend along the entire lateral membrane (white arrows) or are absent altogether (yellow arrows). The percentages indicate the ratio of egg chambers containing partial posterior clones in a monolayer that display this defect. The number of mutant cells in each egg chamber was at least five. All merged images show DAPI in blue, and Fas3, Dlg, Cora or Fas2 in red. *α-Spec* FCCs lack GFP.

All four proteins are expressed uniformly along the lateral membrane in early FE (Fig. 4A-D). From S7, an apicolateral concentration of these proteins becomes distinct in PFCs. This apicolateral localization has been previously observed for Fas3, and our findings show Dlg, Cora and Fas2 to behave similarly. These observations are consistent with transmission electron micrographs that detected incipient SJs at S6 (Müller, 2000), and suggest that the loss of apicolateral accumulation of Fas3 in *α-Spec* FCs might be related to defects in SJ formation. In fact, the apicolateral localization of Fas3, Dlg, Cora and Fas2 is absent in *α-Spec* cells (Fig. 5), even when the mutant cells form a monolayer. It is unclear at this resolution how the SJ components localize in mutant cells; the proteins sometimes extend along the lateral membrane (white arrows) or are absent altogether (yellow arrows). However, a departure from the normal apicolateral accumulation is clear, and it suggests a function for α-Spec in SJ formation.

These localization defects are specific to SJ proteins, and not to all apical junctions, as the adherens junction markers Armadillo (Arm) and E-Cadherin (Shotgun – FlyBase) are largely unaffected in *α-Spec* FCs (Arm 87.5% as control, *n*=16; E-Cadherin 93% as control *n*=14; Fig. S2E-I; data not shown).

### *α-Spectrin* mutant cells fail to form a columnar epithelium

**Fig. 6.**
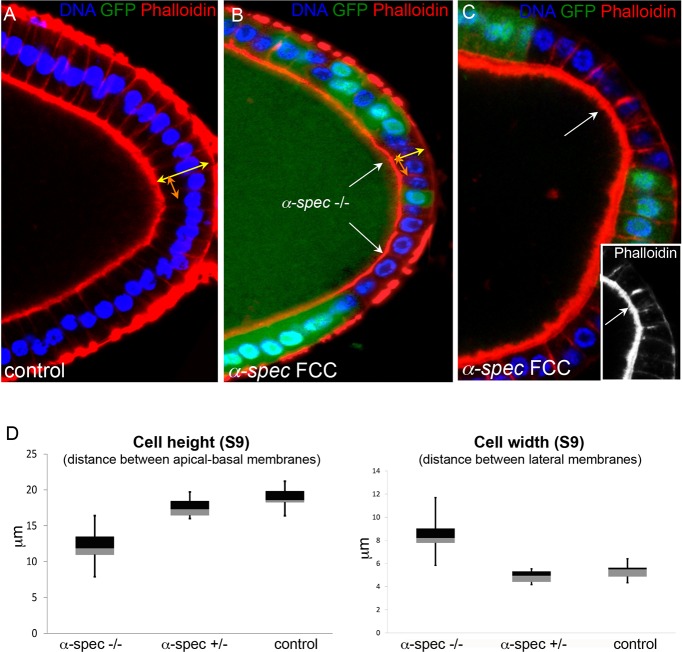
***α-Spectrin* mutant follicle cells fail to form a columnar epithelium.** (A) Most FCs expand their lateral membrane at mid-oogenesis, changing shape from cuboidal to columnar (see also Figs S1 and S4). (B,C) Contrary to wild-type FCs, *α-Spec* cells (*α-Spec* FCCs, *α-Spec*^−/−^) do not extend properly their lateral membrane and fail to become columnar. (C) F-actin levels appear to be higher in *α-Spec* mutant cells. A-C show posterior FCs. DAPI in blue, Phalloidin in red. All *α-Spec* cells lack GFP. White arrows indicate the mutant clone. (D) Box plot quantification at S9. The mean height (yellow arrows in A,B) of wild-type and *α-Spec* cells is 18.95 and 12 µm, respectively. The mean width (orange arrows in A,B) of wild-type and *α-Spec* cells is 5.30 and 8.44 µm, respectively. The Welch two-tailed *t-*test *P*-values between wild type and *α-Spec* mutant (−/−) for cell height and width are 1.123×10^−06^ and 0.0002, respectively. Measurements were performed in posterior cells that maintain a monolayer. *n*=40 (four cells in ten egg chambers) for all samples.

We noticed that *α-Spec* FCCs appear to have a reduced apical-to-basal length (Fig. 5A). Regulation of epithelial cell shape, such as changes in relative sizes of apical, basal and lateral membranes, is key to morphogenesis. FCs undergo various morphogenetic changes, including the transformation from a cuboidal to a columnar epithelium at mid-oogenesis (Dobens and Raftery, 2000). PFCs increase their height four times from S4/5 to S9, but their width remains constant (Fig. S1; Fig. S3A), supporting previous conclusions that FC columnarization does not result from a decrease in apical surface, but rather from an expansion of the lateral membrane, probably driven by cellular growth (Kolahi et al., 2009). To test whether columnarization is aberrant in *α-Spec* FCCs, we measured the height and width of S9 *α-Spec* PFCs. The mean height of control S9 FCs is 18.95 µm, whereas that of *α-Spec* S9 FCs is 12 µm (Fig. 6; *n*=40), suggesting that *α-Spec* mutant cells fail to become columnar. By contrast, the width of *α-Spec* S9 cells is similar to control cells (Fig. 6). Thus, at mid oogenesis, *α-Spec* cells maintain a cuboidal shape, whereas control cells undergo columnarization by growing longer lateral membranes.

The failure of *α-Spec* FCCs to form a columnar epithelium is not a consequence of the tissue architecture being disrupted, as the above measurements were performed in mutant monolayers. Our findings show that SBMS is required for the control of cell shape not only in erythrocytes, but also in epithelial cells. Furthermore, both cell shortening and aberrant localization of SJ components are cell-autonomous defects of *α-Spec* FCCs within a monolayered epithelium, thus possibly preceding the onset of multilayering.

### Similarities between *α-Spectrin* and *integrin* mutant follicle cells

The phenotype of *α-Spec* mutant FCs resembles loss of integrin function. Cells mutant for *myospheroid* (*mys*), which encodes for the only β-chain in the ovary (Devenport and Brown, 2004; Fernández-Miñán et al., 2007), form multilayers, and display differentiation and polarity defects at the ectopic layers, but exit mitosis properly. In addition, we have previously shown that integrins regulate cell shape in the wing epithelium (Dominguez-Gimenez et al., 2007). Similarly, *mys* FCs also show shape defects, with a reduced height, but a similar width, compared with control FCs (Fig. S3B). Thus, both integrins and α-Spec are required for apical contraction-independent cell elongation during FE morphogenesis.

We noticed that *α-Spec* mutant FCs accumulate more F-actin (Fig. 6C, inset; Fig. S4A,B). The actomyosin cytoskeleton has been repeatedly linked to cell shape changes. Rho1 and its effector Myosin II regulate apical-basal length of wing disc cells (Widmann and Dahmann, 2009), and FCs mutant for *Rok* (*Rho kinase*) or for the regulatory chain of Myosin II [also known as MRLC or *spaghetti squash* (*sqh*)] present an abnormal shape (Wang and Riechmann, 2007). The spectrin cytoskeleton provides erythrocytes with mechanical properties, and is somehow functionally linked to the actomyosin network in the *Drosophila* eye (Deng et al., 2015). Clear functional interactions also exist between integrins and actomyosin (He et al., 2010), and we have shown here that *α-Spec* and *mys* FCs show similar phenotypes, including aberrant shape. These observations prompted us to test whether integrins and α-Spec interact with the Rho1-myosin pathway during FE morphogenesis.

### Higher Rho1 or Sqh activity, and lower Myosin II activity, increases and decreases *α-Spectrin* epithelial integrity defects, respectively

**Fig. 7.**
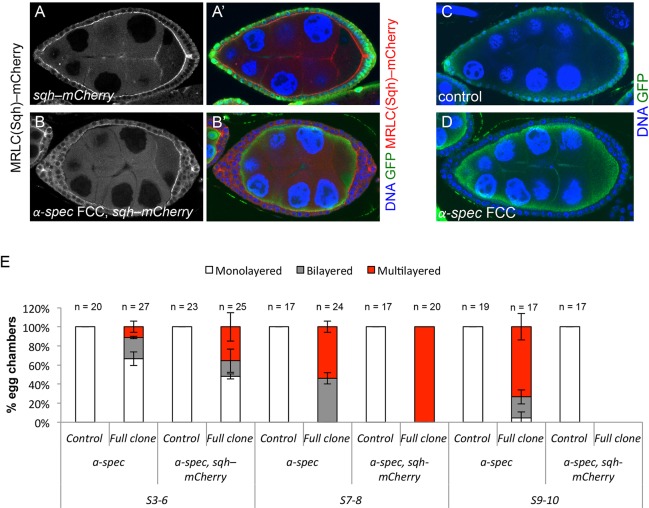
**Overexpressing the Myosin Regulatory Light Chain *spaghetti squash* in egg chambers with *α-Spectrin* mutant clones worsens the multilayering phenotype*.*** (A-D) Sqh-mCherry S8/9 egg chambers without *α-Spec* mutant FCs (A,A′), or with large *α-Spec* clones (*α-Spec* FCCs, B,B′) are compared with egg chambers which contain large *α-Spec* clones only (D). Control egg chambers contain no *α-Spec* clones (A,A′,C). Mutant clones lack GFP. (A-B′) Sqh-mCherry is in red (right panels) or white (left panels). (E) Quantification of the multilayering phenotype reveals a worse defect in egg chambers with large *α-Spec* clones and one extra copy of *sqh* (compare Full clone *α-Spec,sqh-mCherry* with Full clone *α-Spec*). ‘Multilayered’ refers to more than two layers. To simplify the analysis, we only quantified full or very large mutant clones. Cells that express Sqh-mCherry and are heterozygous for *α-Spec* do not show bilayers (control *α-Spec,sqh-mCherry* and A,A′). We did not obtain any S9-10 *α-Spec* egg chambers that also overexpress Sqh. Error bars indicate two different experiments.

To study whether α-Spec might interact with the actomyosin cytoskeleton in regulating FE architecture, we first analyzed the effects of increasing myosin activity in cells that lack α-Spec, using several strategies. Firstly, we overexpressed Sqh (fused to GFP or mCherry) in *α-Spec* FCC-containing egg chambers, and found it to increase S3-6 multilayers from 48% (*n*=25) to 66% (*n*=27) (Fig. 7). This multilayering enhancement is more dramatic at mid-oogenesis, as all S7/8 egg chambers show more than two layers when an extra copy of Sqh was expressed in *α-Spec* cells (*n*=20), compared with 50% when the FCs were only mutant for *α-Spec* (*n*=24) (Fig. 7E; Fig. S5A-C). The fact that all *α-Spec* S7/8 egg chambers display ectopic layers when Sqh was overexpressed prevented us from assessing whether increased myosin enhanced the cell shape phenotype.

Secondly, we increased Rho1 levels in *α-Spec* mutant cells. We were unable to obtain an FE that overexpressed Rho1 (GFP- or mCherry-tagged) and also had *α-Spec* FCCs. Occasionally, we obtained a few cells of the right genotype, but they showed clear signs of undergoing death (data not shown). As an alternative, we overexpressed Rho1 in *α-Spec* mutant cells by driving expression of *UAS-α-SpecRNAi*, *UAS-Rho1* or *UAS-constitutively active-(CA)-Rho1* by the FC-specific driver *trafficjam(tj)-Gal4*. S7/8 egg chambers expressing *α-SpecRNAi* in FCs show multilayers in 33% of the cases (*n*=12), whereas overexpression of *Rho1* or *CA-Rho1* in these cells increases the multilayering phenotype to 50% (*n*=12) and 80% (*n*=10), respectively.

We then tested whether a reduction in myosin activity might rescue the *α-Spec* multilayering. To do this, we reduced the levels of the Myosin II gene *zipper* (*zip*) by driving the expression of *UAS-zipRNAi* with *tj-Gal4*, and inducing *α-Spec* FCCs in the same epithelium. Similarly, we expressed a *zip-dominant negative-(DN)* form in cells that are also mutant for *α-Spec* (Fig. S6). In both cases, we observed a reduction in the S7/8 multilayering phenotype, from 90% (control, *n*=10) to 45% (*zipRNAi*, *n*=11), and from 50% (control, *n*=10) to 20% (*zipDN*, *n*=10).

**Fig. 8.**
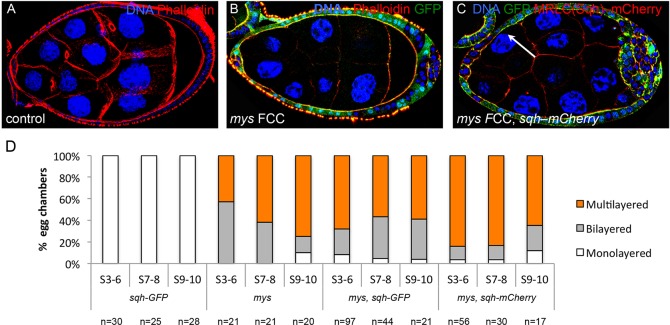
**Over-expressing the Myosin Regulatory Light Chain *spaghetti squash* in *mys* cells enhances the multilayering phenotype*.*** (A-C) S8/9 egg chambers stained for TOPRO (blue, A-C), GFP (green, B,C) and F-actin (A,B, red) or Sqh-mCherry (C, red). Mutant clones lack GFP. (A) Wild type. (B) *mys* mutant clones (FCCs) develop multilayers at the posterior pole. (C) This phenotype is enhanced when an extra copy of *sqh* (*sqh-mCherry*) is expressed in these egg chambers. (D) Quantification of the multilayering phenotype in *mys*, *sqh-GFP*, *mys,sqh-GFP* and *mys*,*sqh-mCherry* egg chambers.

As elimination of integrins affect FCs similarly to the loss of *α-Spec*, we next tested whether integrins might also interact with the actomyosin cytoskeleton to control FE architecture. Indeed, we find that overexpression of Sqh (fused to either GFP or mCherry) in egg chambers containing *mys* mutant FCs increases the percentage of S3-6 follicles with multilayers from 42% (*mys* alone, *n*=21) to 68% (*sqh-GFP*, *n*=97) or 82% (*sqh-mCherry*, *n*=56) (Fig. 8).

To investigate how specific this increased stratification effect of myosin is, we analyzed the impact that overexpressing Sqh has on the multilayering phenotype of *bazooka^815^* FCs. We observe that increasing myosin activity does not enhance *bazooka^815^* multilayers (Fig. S5D-F). All these results together suggest that spectrins and integrins are required to maintain the proper levels of actomyosin activity in the monolayered FE. This is further supported by the fact that both F-actin and Sqh are misplaced and expressed at higher levels in *α-Spec* mutant cells (Fig. 6C; Fig. S4).

### α-Spectrin is required for egg chamber morphogenesis and egg size

Defects in FE tension and morphogenesis are likely to result in egg chambers with aberrant shapes (Wang and Riechmann, 2007). Young egg chambers are spherical, elongating from S5 along the anterior-posterior (AP) axis to create the elliptical shape of the egg. We noticed that *α-Spec* egg chambers seemed rounder than wild-type egg chambers (Figs 1, 3, 7). In fact, S8/9 mutant egg chambers with a multilayered FE either at the anterior, or both anterior and posterior poles, had a shorter AP axis (Fig. S7). This does not seem to be the case when multilayers were only present at the posterior, as the AP axis was not statistically different from controls. Even though mutant egg chambers seem rounder, the dorsoventral axis is slightly shorter in *α-Spec* egg chambers than in controls, suggesting that the total volume of the germline in *α-Spec* egg chambers is smaller.

Egg chambers with integrin mutant FCs render rounder eggs (Bateman et al., 2001). To study whether this is similar in eggs resulting from *α-Spec* mutants, we measured the AP axis in eggs obtained from *α-SpecRNAi*, *tj-Gal4* egg chambers, and observed that, similar to integrins, these eggs also show a reduced AP axis compared with controls (Fig. S7C).

## DISCUSSION

### Revised roles for the spectrin cytoskeleton in regulating proliferation, differentiation and polarity

We find that in the germline α-Spec is not a major regulator of the Hippo pathway. Mutations in *hippo*, *β-Spec* or *α-Spec* result in a stratified FE, but contrary to previous interpretations, and unlike Hippo, spectrins are not required for the FCs to exit mitosis. We believe that the suggestion that Spec mutant FCs over-proliferate was an over-interpretation from the multilayering phenotype, as *α-Spec* cells were not checked for mitotic markers in that report (Fletcher et al., 2015). Again unlike *hippo*, *α-Spec* mutant PFCs only show defects in differentiation when they are located in the ectopic layers of the stratified FE, and oocyte polarity is largely unaffected in mutant egg chambers. It was recently shown that a *β-Spec* allele with a premature stop codon at amino-acid 1046 partially phenocopies *hippo*, with strong defects in FE integrity, actin organization and oocyte polarity (Wong et al., 2015). The null *β-Spec*^G113^ mutant allele (Hulsmeier et al., 2007) behaves similarly to *α-Spec* mutants, showing Hnt defects mainly in ectopic layers (Fig. S8A-B,F), but Fas3 mislocalization in monolayers (Fig. S8C-D′). More importantly, *β-Spec*^G113^ FCCs exit mitosis properly (Fig. S8A,A‴). The differences observed between the two *β-Spec* alleles are likely to be due to the fact that *β-Spec*^G113^ is a null allele.

In conclusion, *α-Spec* and *β-Spec* FCCs do not phenocopy *hippo* mutants when the cells are part of a monolayer, and they seem to adopt a partial *hippo*-like differentiation phenotype only when positioned at ectopic layers, even though *α-Spec* and *β-Spec* cells never divide after S6. Thus, the main function of the spectrin cytoskeleton in FCs is not proliferation control or regulation of the Hippo pathway, although an interaction between spectrins and Hippo might occur once the FCs are within an aberrantly organized FE. The function of spectrins in FCs is in contrast with other tissues, where α- and β-Spec appear to regulate growth through Hippo (Deng et al., 2015; Fletcher et al., 2015; Wong et al., 2015).

### *α-Spectrin* and *β-Spectrin*, but not *β_H_-Spectrin*, mutant epithelia fail to maintain a monolayered architecture

Similar to Hippo, α-Spec and β-Spec are required for the FE to maintain a monolayer. There is an increase in the multilayering phenotype in egg chambers with large clones from S3/6 to S7/8 [36% (*n*=37) and 100% (*n*=34), respectively; Fig. S9A-B]. Also, the presence of control cells in *α-Spec* mosaic epithelia aids the mutant cells to maintain a monolayer from S6, as there is a higher percentage of S7-9 egg chambers with multilayers when the FE contains large *α-Spec* clones (Fig. S9C, ‘full’; 95%, *n*=54) than when the mutant clone is only at the posterior end (Fig. S9C, ‘post’; 53%, *n*=49). The control of FE architecture appears to be mediated by the lateral spectrin network. Loss of α-Spec seems to disrupt both lateral (α/β) and apical (α/β_H_) SBMS in the FE, as β and β_H_ subunits are no longer localized laterally and apically in *α-Spec* cells (Fig. S8G; Lee et al., 1997), but no multilayering was reported for *β_H_-Spec* egg chambers, in which a loss of apical α-Spec was observed (Zarnescu and Thomas, 1999), suggesting that the loss of the lateral α/β is responsible for the FE stratification. Also, β_H_-Spec is mislocalized in *sosie* mutants, but the FE architecture is maintained (Urwyler et al., 2012).

### A novel function for α-Spectrin in localizing septate junction components

Incipient SJs are first detected between the FCs with the completion of proliferation at S6 (Mahowald, 1972; Müller, 2000; this work). We show here that the localization of several SJ components is affected in *α-Spec* FCCs, suggesting that spectrins are required for proper SJ formation. This is further supported by other observations. First, Fas3 localization is affected in *β-Spec* FCCs. Second, Neuroglian (an SJ component) is required for maintaining the stability of the FE (Wei et al., 2004). Third, the reduction of both α- and β-Spec leads to mislocalization of Dlg, Neuroglian and Fas2 in neuromuscular junctions (Featherstone et al., 2001; Pielage et al., 2005). And fourth, it has been suggested that the SBMS and ankyrin associate with SJ components (Bennett and Chen, 2001; Dubreuil et al., 2001).

As the mislocalization of SJ components in Spec mutant FCCs is observed in monolayers, and thus prior to the onset of stratification, we speculate that Spec-dependent distribution of SJ components might contribute to the Spec function in the epithelium. This idea is supported by Crumbs overexpression, which leads to defects in SJs and ZA, and multilayering of the ectoderm cells (Klebes and Knust, 2000; Wodarz et al., 1995), and by *dpak* (Pak – FlyBase) FCs, which mislocalize Fas3 and show multilayering and columnarization defects (Conder et al., 2007). Furthermore, the aberrant accumulation of Fas2 at the lateral membrane of *Tao* FCs prevented membrane shrinking in the cuboidal-to-squamous transition (Gomez et al., 2012). However, *fas3*, *fas2* and *cora* mutant cells do not show shape defects or multilayering (data not shown). Thus, if SJ components contribute to the *α-Spec* phenotype at all, it might be not because they are absent in *α-Spec* mutant cells, but because they are not properly distributed.

### A novel function for α-Spectrin in apical constriction-independent cell elongation

Transitions between squamous, cuboidal and columnar epithelial cell shapes are common during development, and contribute to the morphogenesis of tissues. Here, we demonstrate a cell-autonomous role for α-Spec in promoting the cuboidal-to-columnar shape transition of the FCs. It is important to point out that the FE undergoes lateral elongation without apical constriction (Kolahi et al., 2009; Fig. S3), which might allow phenotypes to be interpreted in a simpler manner. This morphogenetic FC behavior is similar to that of vertebrate neuroepithelia, where cell elongation precedes apical constriction (Suzuki et al., 2012), and it would be interesting to study the function of Spec in the columnarization of these cells.

Although the molecular mechanism of apical constriction-independent cell elongation is unknown, we think that a primary role for the SBMS lies in facilitating changes in cell shape, which is further supported by the cell shape defects in *α-Spec* gut epithelia (Lee et al., 1993), perhaps by contributing to the proper distribution of adhesion molecules. This function of the SBMS in membrane biology is conserved in other cells, as spectrins stabilize the plasma membrane during blastoderm cellularization (Pesacreta et al., 1989), and control photoreceptor morphogenesis through the modulation of membrane domains (Chen et al., 2009; Williams et al., 2004). The spectrin cytoskeleton might also impact on FE columnarization by interacting with the actomyosin cytoskeleton. It is known that apical-basal elongation in drebrin E (drebrin 1) depleted human Caco2 cells is impaired, as a possible consequence of the lack of interaction between drebrin E with spectrins and actomyosin (Bazellieres et al., 2012). Also, the elongation of neuroepithelial cells depends on the assembly of an actomyosin network in the apical junctional complex, regardless of whether cells are constricting or not (Hildebrand, 2005). In *Drosophila* wing discs, the Rho1-Myosin II pathway at the apicolateral membrane seem to regulate the cuboidal-to-columnar shape transition, whereas in the germline, *Rok* and *sqh* mutant FCs fail to adopt a normal shape (Wang and Riechmann, 2007). Finally, SBMS seems to modulate cortical actomyosin contractility in the eye (Deng et al., 2015), and possibly in the FE (this work; Wong et al., 2015). Together, these data suggest that Myosin II activity is aberrant in *α-Spec* mutant FCs, contributing to defects in columnarization and FE architecture.

### Integrins, spectrins and the actomyosin cytoskeleton

Increasing Rho1 and Sqh activities enhances the Spec multilayering phenotype, whereas reducing Myosin II activity decreases it. In addition to this functional link between the SBMS and the Rho-Myosin pathway, we also show that *mys* cells fail to columnarize, and that an extra copy of *sqh* increases the *mys* multilayering phenotype. It has been shown that integrins regulate the Rho-Myosin pathway to induce actomyosin-generated forces (Geiger et al., 2009). Thus, as is the case for spectrins, integrins might also control cell shape and epithelia morphogenesis by modulating the actomyosin activity.

How the SBMS and integrins might modulate actomyosin is unknown, and one possible mechanism is by regulating Myosin II activity directly. However, we would like to propose an alternative mechanism. Spectrins can bind F-actin, and integrins and spectrins interact with proteins involved in the association of F-actin with the membrane (Beaty et al., 2014; Médina et al., 2002). Furthermore, α-Spec and integrins regulate the actin cytoskeleton through Rac (Bialkowska et al., 2005). Previous studies have shown that both *β-Spec* and *mys* mutant FCs display similar defects in the basal level of F-actin (Delon and Brown, 2009; Wong et al., 2015), which are recapitulated in *α-Spec* mutant cells (Fig. S4A). Thus, any defects in actin organization in *mys* and Spec mutant FCs could in turn result in defects in the activity of Myosin II.

Regardless of whether integrins and spectrins regulate F-actin or myosin, or both, spectrins and integrins might act together. The SH3 domain of α-Spec interacts with Tes (Rotter et al., 2005), a component of integrin-dependent focal adhesions (Coutts et al., 2003), and mammalian αII-Spec stabilizes β3-integrin anchorage, suggesting α-Spec as a physical link between focal adhesions and F-actin (Ponceau et al., 2015). In the FE, we observe that α-Spec and αPS1 colocalize in the lateral, and possibly apical, membrane (Fig. S10). In addition, we show that the localization of α-Spec in *mys* clones, and the localization of βPS in *α-Spec* mutant clones, is majorly unaffected (Figs S11, S12). Furthermore, we find that expression of a constitutively active integrin that reduces multilayering of *mys* FCCs (Fernández-Miñán et al., 2007; Meignin et al., 2007), fails to rescue *α-Spec* multilayers (data not shown). Thus, we would like to propose that α-Spec and integrins act independently of each other, but as part of the same functional complex regulating the actomyosin cytoskeleton and tissue architecture.

### What is important for maintaining a monolayered epithelium?

An early event following oncogenic mutations in an epithelium is the escape of the daughter cells from the monolayered epithelium, forming disorganized masses. Spindle orientation has been linked to tumor-like growth in various tissues, and we find that there is a good correlation between spindle misorientation and ‘tumor-like masses’ at the FE: *hippo*, *mys* and *α-Spec* FCCs show misaligned spindles and severe multilayering (Fig. S13; Fernández-Miñán et al., 2007; Meignin et al., 2007), whereas *Notch* FCCs, which overproliferate, do not show multilayering or spindle orientation defects (data not shown). However, perpendicular divisions alone are insufficient to promote stratification, and a mechanism, depending on lateral cell-cell adhesions, is in place to avoid multilayering as a sole consequence of spindle misorientation (Bergstralh et al., 2013, 2015). We would like to propose that spindle misorientation contributes to FE disorganization, but that this ‘safeguard’ mechanism is somehow inactive in *hippo*, *mys* and Spec mutant FCCs. What other aspect of the mutant phenotypes might then be linked to multilayering? A clue might come from the Spec mutant and *mys* FCCs. First, there is an increase in the *α-Spec* multilayers after S6, when both FCs and egg chambers undergo various morphogenetic changes. Second, the volume of the germline surrounded by large *α-Spec* FCCs appears smaller. And third, Myosin II activity is increased in *α-Spec* and *mys* mutant cells. In our interpretation of the results, a proper distribution of Myosin II activity in a Spec- and integrin-dependent manner allows the right amount of forces to be distributed across the membrane and the epithelium. Thus, it is possible that proper cell-cell interactions, adequate force balance and precise spindle orientation are key to maintaining a monolayered epithelium, especially upon the mechanical stress induced by morphogenesis.

## MATERIALS AND METHODS

### Fly stocks and creation of follicle cell clones

*FRT2A* *α-Spec*^*rg41*^ : *α-Spec*^*rg41*^ is an allele containing a 20-base pair deletion that creates a frame-shift and premature termination near the 5′-end of the transcript, and is a null allele (Fig. S1C-D). The original study by Lee et al. (1997) created excision clones of a *p[>lacZ,α-spec>]* construct in flies transheterozygous for the *l(3)α-spec^rg41^* chromosome and the deficiency *Df (3L)R-R2*. This deficiency eliminates *α-Spec*, but also *dlt*, which encodes a protein required for FE formation and polarity (Bhat et al., 1999; Klebes and Knust, 2000). Because of this, heterozygosis of *dlt* in the cells that lack the *p[>lacZ,α-spec>]* construct might have influenced the described phenotypes, and these phenotypes might not be only due to the lack of *α-Spec*.

*sqh-GFP* and *sqh-mCherry* are fusion proteins expression of which is driven by the endogenous *sqh* regulatory sequences and can substitute the *sqh* gene (Martin et al., 2009; Royou et al., 2004). Stocks used were: *FRT42Dhippo^42-47^* (Wu et al., 2003*), FRT19Aβ-spec^G113^* (Hulsmeier et al., 2007), *FRT101mys^11^* (also known as *mys^XG43^*) (Bunch et al., 1992), *FRT19Abazooka^815^* (Djiane et al., 2005), *FRT40Fas3^A142^* (Wells et al., 2013), *FRT19AFas2^G0336^* (Szafranski and Goode, 2007), *FRT42Dcora^1^* (Laprise et al., 2009), *UAS-Rho1* (Bloomington-7334), *UAS-constitutively active-CA-Rho1* (Bloomington-8144), *UAS-zip^RNAi^* (VDRC7819), *UAS-zipDN* (Monier et al., 2010), *UAS-α-spec^RNAi^* (VDRC25387).

To generate most follicle cell mutant clones, we used the heat shock flipase (hs-flp) system (Chou and Perrimon, 1992). Mutant clones were marked by the absence of GFP or RFP (Xu and Rubin, 1993). The heat shock was performed at 37°C for 2 h over 3 days during third instar larval. To generate *mys* and *baz* follicle cell clones, we used the FRT/FLP technique combined with the Gal4 system. The *e22c-Gal4* driver is expressed in the follicle stem cells in the germarium and was used in combination with *UAS-flp*.

The Sqh experiments in Figs 7 and 8 and Fig. S5 used flies that contain three copies of the *sqh* gene: two endogenous ones and the fluorescently tagged version, which is expressed under the *sqh* promotor.

All UAS constructs were expressed by the FC-specific driver *traffic-jam-Gal4* (DGRC104055), except for the *UAS-zipDN* experiment, which was expressed by the MARCM system (mosaic analysis with a repressible cell marker). In this case, hs-flp expression was induced by heat-shocking only adults, at 37°C for 1 h, over 2 days. GFP-positive cells were either *α-Spec* mutant or *α-Spec* mutant overexpressing *zipDN-GFP*.

All females analyzed were between 3 and 10 days old.

Full names of genotypes shown in each figure are listed in supplementary Materials and Methods.

### Antibodies

Primary antibodies were: rabbit anti-β-Spec (1:500; gift of Dr Klämbt, University of Münster, Germany), mouse anti-Fas2 [1:100; 1D4, Developmental Studies Hybridoma Bank (DSHB)], chicken anti-GFP (1:2000; ab13970, Abcam), mouse anti-Arm (1:200; N2 7A1, DSHB), rat anti-E-Cadherin (1:200; DCAD2, DSHB), mouse anti-α-Spec (1:250; 3A9, DSHB), rat anti-Tubulin (1:500; MAB 1864, Chemicon), rabbit anti-aPKC (1:1000; C-20, Santa Cruz Biotechnology), mouse anti-Dlg (1:500; 4F3, DSHB), mouse anti-Fas3 (1:100; 7G10, DSHB), mouse anti-Hnt (1:15; 1g9-s, DSHB), rabbit anti-PH3 (1:500; 06-570, Upstate Biotechnology), rabbit anti-Staufen (1:3000; gift of Dr St Johnston, Gurdon Institute, University of Cambridge, UK), mouse anti-integrin βPS (1:50; CF.6G11, DSHB), mouse anti-Cora (1:100; gift of Dr Gardiol, Gurdon Institute, University of Cambridge, UK). Species-appropriate AlexaFluor488-, AlexaFluor568- and AlexaFluor647-conjugated secondary antibodies were used (1:100; Molecular Probes).

### Immunohistochemistry

For immunostaining, we followed standard procedures (Williams et al., 2014). For F-actin staining, Alexa-coupled Phalloidin (1:200; Invitrogen) was added in PBS/2% Tween-20 for 30 min prior to final washes and mounting. The samples were mounted in Vectashield (Vector)+DAPI, or incubated with the DNA dye TOPRO-3 (1:1000; Molecular Probes) for 10 min and then mounted in Vectashield (Vector).
